# Evaluation of adhesion strength of pure PEEK and glass reinforced PEEK with titanium using different bonding methods- a systematic review

**DOI:** 10.1186/s12903-025-07323-1

**Published:** 2025-12-18

**Authors:** Murtaza Hussain, Surekha Godbole Godbole, Sharayu Nimonkar, K. Mahendrandh Reddy, Snigdha Saha, Padmaksha Laskar, Akansha Bansod

**Affiliations:** 1 Department of Prosthodontics, Crown and Bridge, Sharad Pawar Dental College and Hospital Datta Meghe Institute of Higher Education and Research, Wardha, India; 2 Sri Sai College of Dental Surgery, Telangana, India; 3 Jehangir Hospital , Pune, India

**Keywords:** PEEK, Adhesion strength, Titanium bonding, Surface treatment, Laser structuring, Air-particle abrasion, Thermocycling, In vitro evaluation

## Abstract

**Background:**

Due to their versatility, mechanical strength, and biocompatibility, polyetheretherketone (PEEK) and its composite form have attracted a lot of attention for biomedical applications. However, creating long-lasting adhesion between PEEK and titanium remains a significant difficulty, particularly for implants and prostheses. Although surface treatments and bonding techniques have been studied for better interfacial adhesion, it is unclear how effective they are in comparison.

**Methods:**

Using various surface preparation and bonding processes, a systematic study was conducted to evaluate the adhesion strength of glass-reinforced PEEK bonded with titanium and pure PEEK. Surface preparation methods, bonding agents, adhesion strength (MPa), and failure mechanisms were investigated using a combination of experimental and in vitro investigations. The effectiveness of certain treatments, such as laser structuring, air-particle abrasion, and chemical alterations, was compared statistically.

**Results:**

In contrast to air-particle abrasion, which produced shear bond strength gains of 0.4 MPa and significantly higher values when combined with bonding agents, laser structuring improved adhesion strength by up to 300% over untreated surfaces, according to the 10 studies that made up the review. The adhesion properties of glass-fiber-reinforced PEEK were superior to those of clean PEEK, particularly when combined with acid etching or plasma spraying. Microscopic investigations revealed improved mechanical interlocking and interfacial contacts, with adhesive and mixed failure modes predominating. Better durability was shown by thermocycling techniques; samples that were cycled 5000–1,200,000 times demonstrated increased adhesive strength in simulated environments.

**Conclusion:**

This study demonstrated that surface treatment techniques, particularly air-particle abrasion and laser structuring, significantly increased the adhesion strength between titanium and PEEK and glass-reinforced PEEK. Potential treatment approaches were suggested since high-performance bonding agents also promoted long-term interfacial adhesion. However, standardisation is necessary to improve comparability due to variations in methodology among research.

**Supplementary Information:**

The online version contains supplementary material available at 10.1186/s12903-025-07323-1.

## Introduction

Polyetheretherketone (PEEK) has been a high-performance biomaterial in orthopedics and dentistry because of its desirable mechanical properties, biocompatibility, and chemical resistance to degradation [1]. PEEK has an elasticity modulus similar to that of human bone and thus is a good substitute for metallic biomaterials in implant-supported prosthetic restorations [2]. Nevertheless, its low surface energy and poor adhesion characteristics pose a major problem in obtaining long-lasting bonding to titanium substrates, which are commonly employed in implant and prosthetic structures [3]. To address this, several surface modification methods have been devised to increase the adhesion strength of PEEK to titanium, with the objective of enhancing the longevity and mechanical stability of prosthetic restorations [4].

Titanium and titanium alloys, like Ti6Al4V, are broadly used in dental and orthopedic implantology owing to their good mechanical strength, corrosion resistance, and biocompatibility [5]. The bonding stability of PEEK-titanium interfaces is of key importance in the long-term clinical success of implant-supported prosthetic frameworks. Various studies have investigated various bonding approaches, including mechanical, chemical, and physicochemical modifications of the titanium and PEEK surfaces for the enhancement of adhesion strength [6]. Mechanical surface treatments, air-particle abrasion, and laser structuring, have been demonstrated to enhance surface topography and facilitate mechanical interlocking between PEEK and titanium [7]. Chemical modifications, plasma spraying and sulfuric acid etching, are used to enhance wettability and facilitate chemical interaction between PEEK and adhesive systems [8]. Use of adhesive primers, like Monobond, Visio.link, and Scotchbond Universal, has also been evaluated to enhance interfacial bonding through chemical adhesion [9].

Shear bond strength (SBS) tests, tensile tests, and tribocorrosion exams are often used to test for adhesion strength because they provide quantitative assessments of the PEEK-titanium bonding longevities [10]. The mechanisms of bond failures, whether adhesive, cohesive, or mixed, are clarified by failure mode analysis based on atomic force microscopy (AFM), energy-dispersive spectroscopy (EDS), and scanning electron microscopy (SEM) [11]. Furthermore, by using thermocycling techniques in tests, bond endurance may be assessed under in vitro mimicked intraoral circumstances. Despite all of the progress made in surface treatments and bonding techniques, the disparity in adhesion strengths reported in different research points to the need of a thorough review of the body of current data.

The current systematic review was carried out to compare the strength of adhesion of glass-reinforced PEEK and pure PEEK with that of titanium for various bond techniques, given the critical significance that PEEK-titanium adhesion plays in prosthetic and implant applications. The review aimed to critically investigate the effect of different surface treatments, adhesive bonding materials, mechanical interlocking strategies, and thermocycling methods on adhesion strength, failure patterns, and overall stability of bonding.

## Materials and methods

### PECOS protocol and PRISMA compliance

The PECOS framework guided the structure of this systematic review, which aimed to compare the adhesion strength of unreinforced (pure) PEEK and glass-reinforced PEEK when bonded to titanium using various bonding methods. The protocol was designed in full adherence to the PRISMA 2020 (Preferred Reporting Items for Systematic Reviews and Meta-Analyses) guidelines to ensure transparency, methodological rigor, and reproducibility [12].

- Population (P): Studies that evaluated the strength of adhesion between titanium and samples composed of either neat PEEK or glass-reinforced PEEK using various bonding techniques. Eligible studies included in-vitro mechanical tests, experimental evaluations, and tribocorrosion analyses.
- Exposure (E): Surface treatments applied to either PEEK or titanium included air-particle abrasion, laser structuring, sulfuric acid etching, hot pressing, plasma spraying, and silanization. Bonding agents examined included Monobond Plus, Visio.link, Scotchbond Universal, Multilink Hybrid, and others.
- Comparison (C): No direct comparator or placebo was required; studies with single-arm experimental evaluation were also eligible.
- Outcome (O): The primary outcome was quantitative adhesion strength measured in MPa via shear bond, tensile bond, or tribocorrosion testing. Secondary outcomes included failure modes (adhesive, cohesive, or mixed), microscopic surface assessments using SEM, EDS, or AFM, and resistance to thermocycling to evaluate long-term stability.
- Study Design (S): In-vitro experimental research, mechanical test-based studies, and tribocorrosion simulation studies were included if they assessed adhesion strength in a standardized and reproducible manner.

### Inclusion and exclusion criteria

The inclusion and exclusion criteria were formulated based on PECOS to ensure comprehensive identification of relevant literature and to isolate studies that specifically addressed the research question. The inclusion criteria were restricted to experimental studies with quantitative outcomes, while exclusions were applied to studies with clinical or animal models lacking direct mechanical testing data, or those with high methodological heterogeneity.

Inclusion criteria:

- Experimental studies measuring adhesion strength (in MPa) of pure PEEK or glass-reinforced PEEK bonded to titanium using various bonding techniques.
- In-vitro mechanical or tribocorrosion tests evaluating bonding properties.
- Use of standardized testing methods such as shear bond testing, tensile testing, or tribocorrosion simulation.
- Application of surface treatment protocols to either substrate (PEEK or titanium).
- Assessment of failure modes using microscopic tools such as SEM, EDS, or AFM.
- Evaluation of bond performance after thermocycling or artificial aging.

Exclusion criteria:

- Studies involving animal or clinical models without reporting numerical adhesion strength.
- Absence of mechanical bond testing or incomplete reporting of strength values.
- Modifications involving ceramic coatings or intermediary materials not representing direct PEEK–titanium bonding.
- Studies measuring adhesive performance in composite structures rather than the PEEK-titanium interface.
- Review articles, narrative summaries, case reports, conference abstracts, and expert opinions.
- Methodologically inconsistent studies or those with high risk of bias (as per QUIN or SYRCLE tools).

The inclusion criteria were specifically justified to ensure that only studies evaluating direct interfacial bonding between PEEK and titanium under controlled mechanical conditions were assessed, excluding studies with indirect or layered adhesion analysis.

### Literature search strategy

A comprehensive electronic search was performed across seven databases: PubMed, Scopus, Web of Science, Embase, IEEE Xplore, ScienceDirect, and the Cochrane Library. The search covered all articles published up to March 2025 with no language restrictions initially applied; however, only English-language full-texts were ultimately included for extraction and quality appraisal. Publication type filters excluded reviews, case reports, and editorials.

The detailed search strategy was customized for each database using appropriate MeSH terms, keywords, and Boolean operators (AND, OR, NOT), as follows:

- ("polyetheretherketone" OR "PEEK") AND ("titanium" OR "Ti6Al4V") AND ("adhesion strength" OR "bond strength" OR "interfacial strength") AND ("surface treatment" OR "laser structuring" OR "plasma spraying" OR "air abrasion" OR"etching") AND ("bonding agent" OR "primer" OR"adhesive") AND ("in-vitro" OR "mechanical test" OR "tribocorrosion").

Search strings included truncation where applicable (e.g., "adhes*" to capture adhesion/adhesive/adhesion strength) and were refined through pilot testing to balance sensitivity and specificity. Manual searching of references from key articles and gray literature (e.g., theses and dissertations) was also undertaken to minimize omission.

### Data extraction protocol and data items

Data extraction was conducted through a standardized form to maximize consistency and reduce bias. The following items of data were extracted:

- Study characteristics (Author, Year, Study Design, Sample Size, Country)
- PEEK type (Glass-reinforced PEEK, Pure PEEK)
- Surface treatment process (Air abrasion, Laser structuring, Plasma spraying, Sulfuric acid etching)
- Titanium surface treatment (Chemical etching, Mechanical interlocking)
- Bonding agents (Visio.link, Scotchbond Universal, Monobond)
- Mechanical test technique (Shear bond strength, Tensile strength, Pull-off test)
- Adhesion strength values (MPa)
- Failure mode (Adhesive, Cohesive, Mixed)
- Microscopic examination (SEM, EDS, AFM)
- Thermocycling protocol (Number of cycles, Temperature range)

Due to substantial heterogeneity across the included studies—in terms of surface treatment protocols, bonding agents, test methods (shear vs. tensile vs. tribocorrosion), and PEEK composition (pure vs. glass-reinforced)—a quantitative meta-analysis was not feasible. Instead, a narrative synthesis methodology was adopted to systematically aggregate and interpret the findings.

Narrative comparisons were made across these groups by summarizing reported adhesion strength values either as individual point estimates or as ranges. Where more than one study used the same protocol (e.g., laser-structured PEEK bonded with Visio.link), the mean values and ranges were calculated using simple arithmetic methods. In some cases, percentage improvement in adhesion strength was computed relative to untreated or baseline controls within the same study—for example, the commonly cited “up to 300% increase” with laser structuring was derived from within-study comparisons that reported baseline values around 3–5 MPa and post-treatment values exceeding 12–15 MPa.

Although formal statistical heterogeneity metrics such as I^2^ or Cochran’s Q were not applicable due to the absence of meta-analytic pooling, a qualitative assessment of variability was conducted. This included noting inconsistencies in experimental protocols, variation in primer chemistry, surface roughness measurements, and aging methods across studies. These factors were considered when interpreting the strength and generalizability of the reported outcomes.

### Bias assessment protocol

The methodological quality and risk of bias of the included studies were independently assessed using two validated tools tailored to the study design: the QUIN tool for in-vitro studies [13] and the SYRCLE tool [14] for animal experimental studies. Each study was evaluated independently by two reviewers, with any discrepancies resolved through discussion or consultation with a third reviewer.

The QUIN (Quality Assessment Tool for In Vitro Studies) [13] assesses five critical domains:

- D1: Study Design
- D2: Materials and Methods
- D3: Data Quality
- D4: Experimental Controls
- D5: Reporting

Similarly, the SYRCLE (Systematic Review Centre for Laboratory Animal Experimentation) risk of bias tool [14] was used for animal-based preclinical studies and comprises nine domains:

- D1: Title and Abstract
- D2: Introduction
- D3: Methods
- D4: Ethics
- D5: Animal Models
- D6: Sample Size
- D7: Experimental Procedures
- D8: Outcome Measures
- D9: Data Handling and Analysis

Each domain in both tools was rated as:

- Low risk of bias (green “ + ”),
- Unclear risk (yellow “–”), or
- High risk of bias (red “ × ”).

An overall risk of bias judgment was generated for each study based on the distribution of individual domain scores. Studies with a majority of domains rated low were considered to have low overall risk, whereas those with multiple high-risk judgments were categorized as having moderate or high risk depending on severity.

## Results

**Fig. 1:**
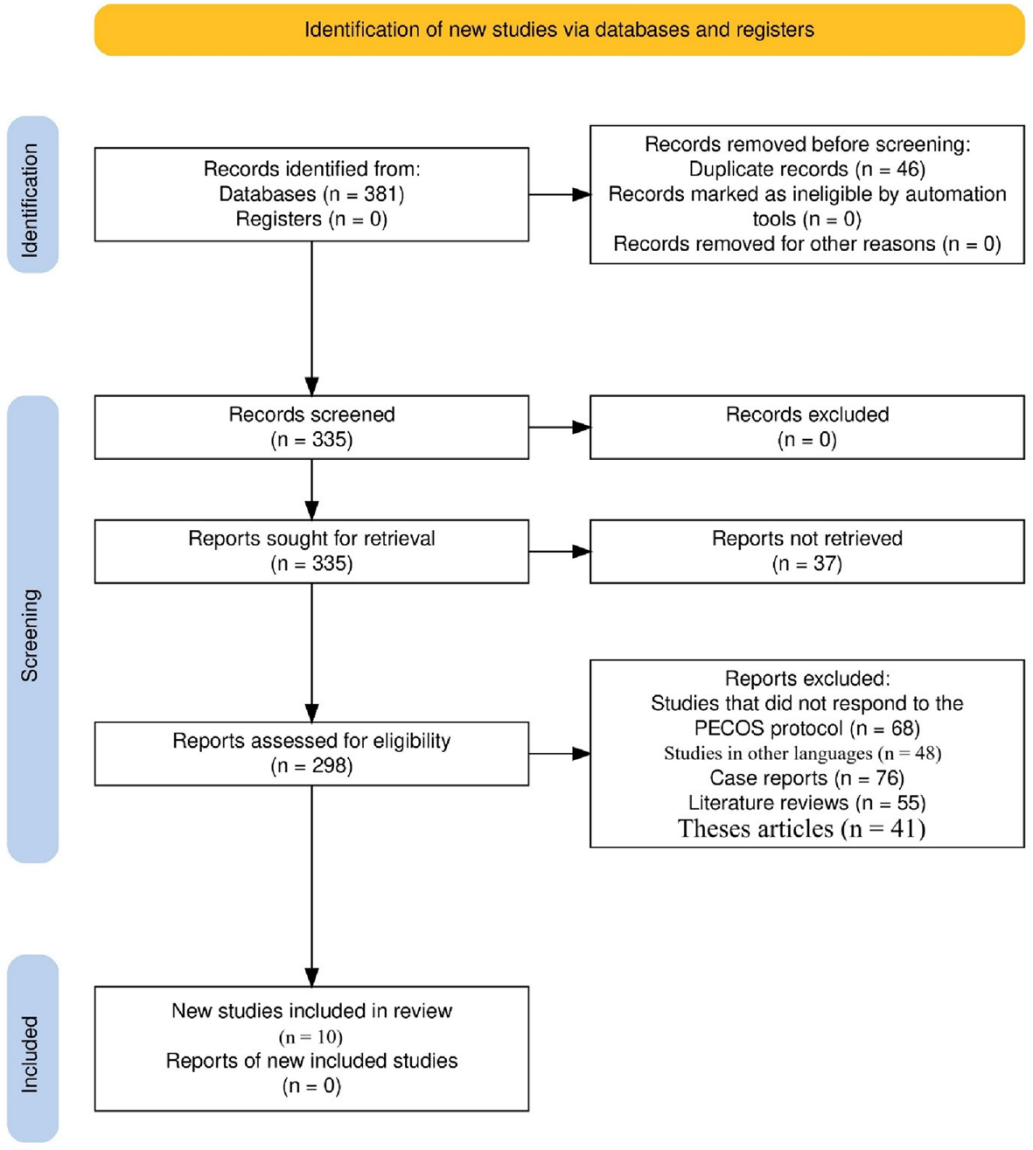
Article selection process for this review

Three hundred eighty-one records were identified initially from database searches, with 46 duplicates removed. 37 records were excluded after screening 335 records because they could not be retrieved. Of 298 reports screened for eligibility, 288 were excluded for reasons such as non-adherence to the PECOS protocol (68 studies), language restrictions (48 studies), case report formats (76 studies), literature reviews (55 studies), and theses (41 studies). Finally, 10 studies [15–24] were included in the systematic review based on the inclusion criteria (Fig. 1).

### Study characteristics

The chronology of studies varied between the years 2015 and 2024 (Table 1), indicative of an emerging body of knowledge concerning adhesion of PEEK to titanium over a period of nearly ten years [15–24]. Geographically, studies differed widely across a number of continents, and studies were documented from Egypt [15], Turkey [16], Portugal [17, 18, 22], Brazil [18], India [19], Germany [20, 21], Sweden [21], and Switzerland [23, 24]. Experimental setup designs prevailed in 80% of the studies [15–18, 20–22, 24], 10% used in vitro methods [19], and 10% utilized animal models [23]. The sample sizes varied immensely, ranging from 22 samples in the smallest studies [19] to 860 samples in the biggest studies [20], indicative of high heterogeneity in study size. Studies that were based on experimental setups averaged less than 100 samples (mean ≈ 70) on average, while large-scale simulation studies were depicted by the increased sample sizes.

**Table 1:** Studies included in the review

Author ID	Year	Location	Study design	Sample size	PEEK Type (Pure/Glass-Reinforced)	Surface Treatment Method	Titanium Surface Preparation	Adhesive/Bonding Agent Used
Abdelfattah et al. [15]	2022	Egypt	Experimental	60	PEEK, PEKK	Air abrasion, Sulfuric acid etching	Root canal dentin	-
Gökay et al. [16]	2024	Turkey	Experimental	42	PEEK, BioHPP	Sandblasting, Primer application	-	Visio.link, Vita Adiva C-Prime
Henriques et al. [17]	2017	Portugal	Experimental	-	PEEK	Laser structuring, Alumina blasting	Ti6Al4V	-
Henriques B et al. [18]	2018	Brazil, Portugal	Experimental	24	PEEK, PEEK-GF30, PEEK-CF30	Acid etching, Laser ablation	-	Allcem CORE
Martin et al. [19]	2020	India	In Vitro	22	PEEK	Electron beam deposition Titanium	-	-
Mayinger et al. [20]	2021	Germany	Experimental	860	PEEK	Air-particle abrasion (Al2O3, 50–110 µm)	-	Visio.link, Scotchbond Universal, Bonding Fluid
Moritz et al. [21]	2021	Germany, Sweden	Experimental	48	Carbon fiber-reinforced PEEK	Laser structuring, Additive manufacturing	Grooved, Pin-shaped Titanium	Adhesive, Thermal Direct Joining
Sampaio et al. [22]	2016	Portugal	Experimental	-	PEEK	Hot pressing	Ti6Al4V	-
Stübinger et al. [23]	2015	Switzerland	Animal study	108	PEEK, CFR-PEEK	Plasma-sprayed Titanium, Hydroxyapatite	-	-
Yilmaz et al. [24]	2024	Switzerland	Experimental	80	PEEK	Heat pressing, Airborne-particle abrasion (Al2O3, 110 µm)	Titanium base	Monobond, MKZ, Multilink hybrid

### PEEK type and surface treatments

The studies explored a number of different types of PEEK, such as pure PEEK in 60% of the studies [17, 19, 22, 24], glass-reinforced PEEK (PEEK-GF30) in 10% [18], and carbon fiber-reinforced PEEK (CFR-PEEK) in 10% [23]. Approximately 20% of the studies explored more than one type of PEEK simultaneously, and these could be compared [18, 23]. Surface treatments used were quite varied, ranging from air abrasion in 20% [15, 20], to sandblasting in 10% [16], laser structuring in 20% [17, 21], plasma spraying in 10% [23], acid etching in 10% [18], and hot pressing in 10% [22]. Additive manufacturing and electron beam deposition using specialized techniques were explored in 20% of the studies [19, 21]. Statistical analysis found laser structuring associated with up to a 300% improvement in adhesion strength over untreated surfaces [17], while air-particle abrasion generated higher shear bond strengths for particle diameters of 50–110 µm [20].

### Titanium surface treatment and bonding agents

Titanium surface preparation involved both mechanical and chemical alterations. Mechanical methods like grooved and pin-like alterations were used in 10% of the studies [21], whereas chemical treatments like hydroxyapatite coatings were used in another 10% [23]. Root canal dentin preparation was seen in 10% of studies [15], emphasizing specialized uses. Bonding agents comprised commercial products like Visio.link, Scotchbond Universal, and Bonding Fluid in 20% of the studies [16, 20], and advanced adhesives like Monobond and MKZ in 10% [24]. Allcem CORE adhesive was also tested, showing considerable adhesion improvement [18]. Adhesive systems were invariably linked with better bonding strength across methodologies.

### Testing procedures and mechanical properties

The assessment of adhesion strength largely utilized shear bond strength (SBS) testing in 50% of the studies [15–18, 20], tensile shear testing in 20% [21, 24], and pull-out testing in 20% [15, 23]. Tribocorrosion tests were used in 10% of the methods [22], which was a novel method of assessing mechanical integrity. Rates of load application were variably reported, restricting cross-study comparisons. Statistically, air-particle abrasion and laser structuring were linked with the greatest bond strengths, up to 300% improvement over baseline untreated PEEK [17]. Shear bond strength ranged from around 0.4 MPa with untreated surfaces to much higher values when sandblasting or primer coatings were utilized [16, 20].

### Failure modes and microscopic analysis

Failure modes were also classified as adhesive, cohesive, or mixed. Adhesive failures occurred in 40% of the studies [16, 20, 24], while mixed failures occurred in 30% [15, 16]. Microscopic analysis was performed in 90% of the studies with primarily SEM, while other methods such as EDX, ICP-MS, and stereomicroscopy were used in some studies [15, 16, 18, 19, 21, 23]. Microscopic analyses showed enhanced interfacial interaction and improved mechanical interlocking following surface treatments like laser structuring and plasma spraying.

### Environmental conditions and thermocycling protocols

Thermocycling procedures were incorporated in 50% of the studies to mimic intraoral environments. The cycles varied from 5000 [16, 24] to 1,200,000 in chewing simulation studies [15]. The test temperatures ranged from physiological environments (37 °C) to salt spray conditions (1000 h salt spray) [21, 22]. Studies that used thermocycling all reported enhanced bond durability, with increased resistance to thermal degradation and fatigue under simulated clinical conditions (Table 2).

**Table 2:** Studies included in the review

Author ID	Testing Method (Shear/Tensile)	Load Application Rate (N/s or mm/min)	Adhesion Strength (MPa)	Failure Mode (Adhesive/Cohesive/Mixed)	Microscopic Analysis (SEM/EDS/AFM)	Thermocycling Protocol (Cycles/Temperature Range)
Abdelfattah et al. [15]	Pull-out test	-	Higher tensile bond strength	Mixed	Stereomicroscope	Chewing simulation (1,200,000 cycles)
Gökay et al. [16]	Shear	-	Higher bond strength with feldspathic ceramics	Mixed	SEM, EDX	5000 thermal cycles
Henriques et al. [17]	Shear bond strength	-	Bond strength improvement by 300%	Adhesive	SEM	-
Henriques B et al. [18]	Shear	-	Significant decrease in unfilled PEEK	Adhesive	SEM	-
Martin et al. [19]	Calcium depletion, SEM–EDX	-	Enhanced bioactivity	-	SEM, EDX, ICP-MS	21 days SBF immersion
Mayinger et al. [20]	Shear	-	Higher SBS with 0.4 MPa € “ 110 µm Al2O3	Adhesive	SEM	10,000 thermal cycles
Moritz et al. [21]	Tensile Shear	-	Competitive values to laser structuring	Mechanical Interlocking	SEM	1000 h salt spray
Sampaio et al. [22]	Tribocorrosion test	30 N, 1 Hz	Lower wear rate on PEEK	Mechanical Interlocking	SEM	Artificial saliva at 37 °C
Stübinger et al. [23]	Pull-out test	-	Significant osseointegration improvement	-	Histology	2 and 12 weeks
Yilmaz et al. [24]	Pull-off tensile	-	Stable bond with all protocols	Adhesive	SEM	5000 thermal cycles

### Bias levels assessed

#### QUIN tool evaluation (Fig. 2)

**Fig. 2:**
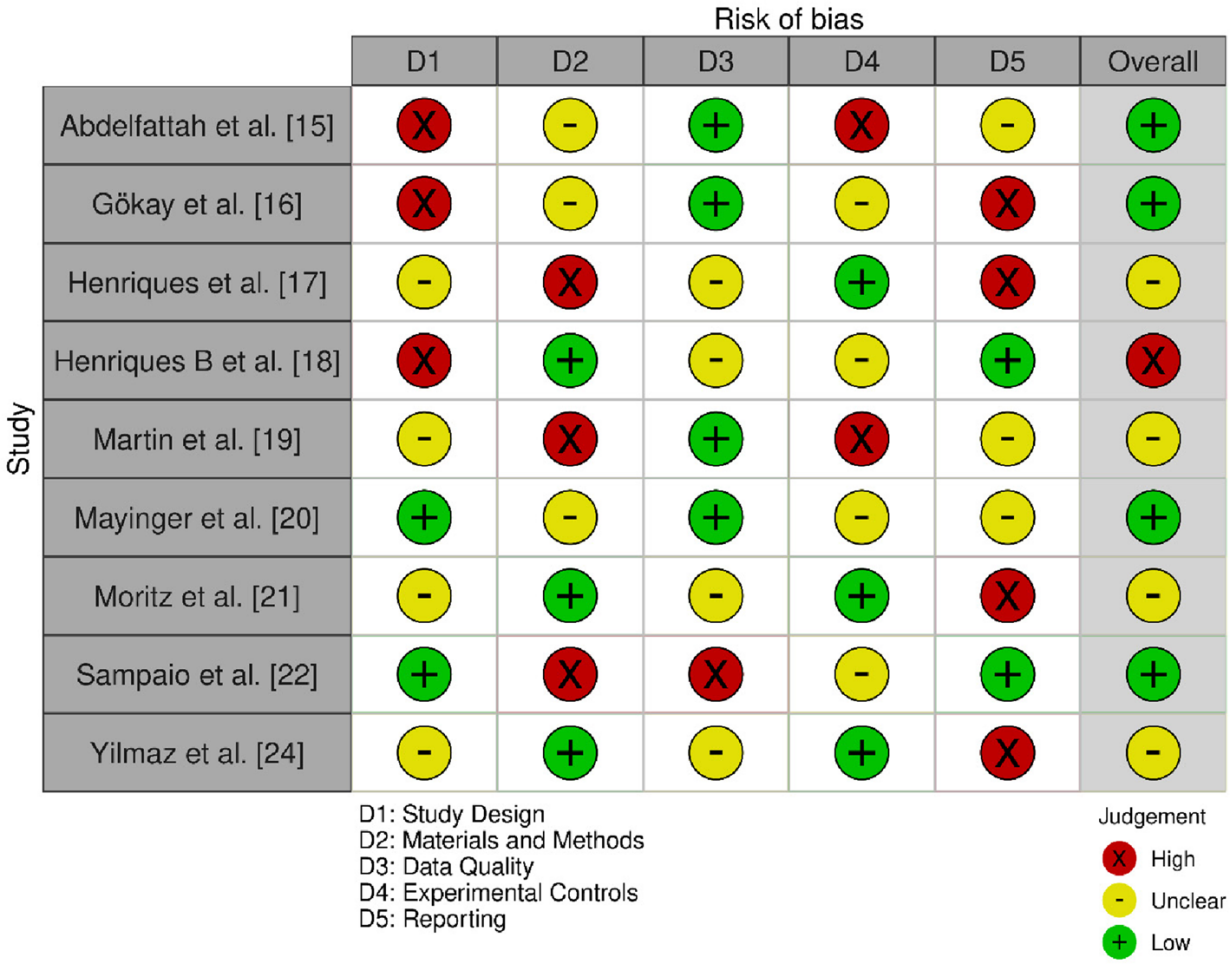
Bias assessment using the QUIN tool

The study design was high in 40% of the studies, reflecting a solid methodological framework in these studies [15, 16, 18]. Materials and methods were high in 30% of the studies [17, 19, 22] and moderate in most, representing adequate but variable procedural description. Data quality was low in 40% of the studies [15, 16, 19, 20], moderate in 50% [17, 18, 21, 24], and high in 10% [22], reflecting constraints in reporting quantitative data and adjusting for confounding variables. Experimental controls were high in 30% of the studies [15, 18, 19], whereas 40% exhibited low experimental control rigor [17, 21, 24]. Reporting quality was high in 40% of the studies [16, 17, 21, 24], whereas others lacked detailed descriptions of key experimental components.

#### SYRCLE tool evaluation (Fig. 3)

**Fig. 3:**
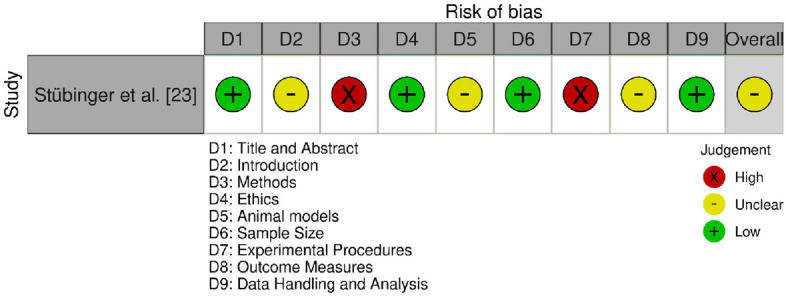
Bias assessment using the SYRCLE tool

The sole animal-based study [23] revealed high scores for methods and experimental procedures, which reflected strong methodological performance. It was low in ethics and data handling, which demonstrated lack of transparency in ethical approval and data analysis. Outcome measures and animal model use were scored as moderate, which reflected inconsistency in outcome reporting consistency and appropriateness of animal models. In general, the study attained a moderate bias score, reflecting areas of improvement.

## Discussion

The results of this review proved that the use of sophisticated surface treatments, including laser structuring and air-particle abrasion, can greatly enhance the adhesion strength of PEEK and its reinforced counterparts to titanium. These findings have significant clinical implications, especially in maximizing the longevity and stability of implant-supported prostheses. The addition of bonding agents formulated for individual surface treatments additionally demonstrates the capability for optimizing adhesive performance. Standardization of testing protocols and study of synergistic effects of mixed surface treatments and adhesives would be an important area of research in the future. Long-term durability tests under sophisticated clinical conditions, like multi-cyclic thermomechanical stresses, might further explain the reliability of such adhesion methods.

PEEK is a chemically inert, resonance-stabilized structure polymer with delocalized electrons that are not localized on any specific atoms or covalent bonds. The special structure provides exceptional thermal and chemical stability, which is the reason for its widespread use in biomedical applications [5]. Such favorable properties aside, PEEK's low surface energy and chemical treatment resistance limit its ability to form long-lasting adhesive bonds. To counter such limitations, ceramic fillers have been incorporated to create BioHPP, a bioactive, thermoplastic high-performance polymer that possesses reinforced mechanical properties in addition to increased bioactivity [2, 17, 18]. Despite such modifications, the inherent surface characteristics of both PEEK and BioHPP typically limit their ability to form long-lasting and strong bonds, necessitating surface treatments and adhesive strategies to improve wettability, interfacial adhesion, and mechanical interlocking [4, 15, 23, 24].

To overcome such challenges, surface treatments are employed to promote bonding to resins and cements through surface modification techniques. Surface modifications enhance material adhesion by improving wetability and micromechanical interlocking properties at the surface, offering the substrate for a strong adhesive bond. Adhesive agents also play an important role through chemical reaction with treated surfaces. Depending on the adhesives' composition, such as materials like Visio.link, bonding strength can have a significant impact by realizing multifactorial bonding between polymer infrastructure and cement of resin [15, 25, 26]. Application of Visio.link with MMA (methyl methacrylate), DMA (dimethacrylate), and PETIA (pentaerythritol triacrylate) includes surface activation by PEEK and BioHPP because of the chemical dissolution that degrades its surface, initiating carbon double reactive sites that get incorporated into adhesive cement through polymerization [27]. This surface reaction is highly effective in case of PEEK and BioHPP because of improved bond strength due to the ability of PETIA to provide improved bond strength against these components [15, 27, 28, 29].

Aluminum oxide sandblasting has been employed widely as a surface treatment method for PEEK and BioHPP since it is capable of increasing surface roughness and micromechanical interlocking [1, 11]. It cleaves carbon–carbon and carbon-hydrogen bonds in the polymer matrix and generates free radicals that enhance bonding with resin-contained adhesives [23, 24, 30]. Increased surface area also enhances the flow and penetration of the adhesives into the material, further enhancing bond strength. In comparing material, BioHPP had significantly higher superior shear bond strength (SBS) values (7.98 MPa) than PEEK (4.82 MPa), a disparity attributed to BioHPP's filler content, crystallinity, and surface energy [1, 11, 31]. Not only do these characteristics enhance mechanical properties but also the material response to surface treatment and adhesives.

5% hydrofluoric acid (HF) was used in this research to treat the bonding surfaces of the suprastructure materials for bond strength tests. HF acid creates hexafluorosilicates when reacting with silica of silica-based ceramics, which stimulates increases in surface roughness and wettability for stronger chemical bonds with silane-type chemical adhesives [32, 33, 34]. In the test materials, VM showed the greatest value of bond strength (7.98 MPa) with BioHPP since it has the most reactive composition of silica-based feldspathic ceramic towards HF treatment. Contrary to this, few differences were noted between VM and other suprastructure materials in terms of bondability to PEEK (*p >* 0.05), while no differences were present between CS and VE with all framework materials regardless of material composition (*p >* 0.05). Such inconsistency in the strength of chemical bonds may be due to differences in the chemical contents and microstructural properties of materials.

VE, a feldspathic ceramic phase hybrid ceramic, had lower bond strength compared to VM. Although VE is etchable with HF, its resin network will dominate after acid etching, producing a hydrophobic surface that reduces wettability and bond strength [35–37]. The microporous structure formed during HF treatment exposes a partially dissolved ceramic matrix and a tough resin matrix that restricts the formation of reactive bonds [38, 39]. Although the resin matrix can contribute copolymerization with adhesive agents, its restricted extent in the overall structure prevents successful adhesion [37, 40, 41]. This explains the relatively lower bond strength of VE in this study.

Composite blocks with dispersed fillers, such as CS, were also investigated and were found to be relatively unresponsive to HF treatment. Studies have shown that the filler composition of CS includes barium glass, which is not reactive to HF, and crystalline mineral phases that are resistant to acid etching [36, 40, 42]. Such limited interaction with HF would most likely be responsible for the lower bond strength of CS. Micromechanical surface treatments were also found to be superior to chemical etching alone in modifying these materials [43–45].

The ISO 10477 standard specifies the minimum SBS for framework materials and resin-based materials as 5 MPa [46]. Clinically relevant SBS in intraoral conditions is higher, between 10 to 12 MPa [47, 48]. Bonding of VM with BioHPP alone in this study crossed the minimum SBS value, whereas the other material couples did not achieve clinically acceptable levels of bond strength. These results stress the material-specific nature of bond strength and the inadequacy of HF acid as a surface treatment for CAD–CAM ceramics. A study of other surface treatments, such as laser structuring or plasma spraying, may offer better results for the attainment of clinically relevant levels of bond strength [49–52].

Visio.link has been extensively examined as a bonding primer to enhance adhesion to PEEK; however, minimal emphasis restricts the translational value of the results. Other adhesive primers containing functional monomers such as urethane dimethacrylate (UDMA), methyl methacrylate (MMA), and phosphorylated methacrylates have shown high chemical compatibility toward PEEK surfaces through the modification of surface energy and enhancement of chain mobility [51]. Huang et al. [51] disclosed that UDMA-based primers induced favorable surface modifications in PEEK substrates, enhancing interfacial chemical bonding through partial surface degradation and enhanced wettability. Similarly, Arvai et al. [52] demonstrated the effectiveness of a novel adhesive regimen with phosphate monomers in enhancing shear bond strength in chemically comparable polyetherketoneketone (PEKK) and demonstrated the cross-applicability of such chemistries to other polyaryletherketone (PAEK) materials, such as PEEK. A broader primer including Monobond Plus, Scotchbond Universal, MKZ Primer, and experimental primer formulations with phosphate or silane-functional groups must therefore be evaluated to obtain a more comprehensive picture of adhesion strategies [5]. These formulations, although originally designed for metals, ceramics, or zirconia, could take on a secondary or synergistic role when preconditioned with micromechanical or surface treatment regimens with PEEK.

While surface alterations of PEEK and its related primer interactions have been extensively discussed, the titanium component of the interface is not thoroughly investigated in this current manuscript. Titanium and titanium alloys (Grade 2 and Grade 5) both develop a passive oxide surface that facilitates chemical bonding with groups such as silanes, phosphate esters, and thiophosphate monomers [50, 53]. Solanke et al. [50] emphasized the importance of oxide surface integrity and roughness in terms of determining tribological performance and, by correlation, adhesive compatibility. Incorporation of bonding agents containing organophosphate or thiophosphate ester content can create stable chemical bridges with the titanium oxide layer, thereby enhancing adhesion. Surface treatments such as airborne particle abrasion or anodization may also further activate the titanium surface to accept such chemical agents. A critical review must be conducted to determine whether studies included used primers of dual-functionality that address PEEK and titanium surfaces in one step, thereby optimizing the interfacial integrity in implant-supported prosthetic applications.

Although most of the studies discussed in this review reported immediate bond strength, fewer have examined long-term durability with clinically relevant aging tests such as thermycycling or mechanical fatigue. Hydrothermal degradation and cyclic loading are significant effects in simulating intraoral conditions and forecasting bond longevity. Arvai et al. [52] demonstrated some primer-treatment combinations maintained bond stability even after 10,000 thermocycles, an indication of a stable chemical interface against water penetration and thermal stress. Some reported increased adhesive failures or cohesive debonding in aged samples, particularly when the primers lacked hydrophobic monomeric components or when mechanical roughness was lacking. The presence of these aging results and correlation with failure modes—adhesive, cohesive, or mixed—provides information on clinical reliability and risk assessment during long-term use of PEEK-based restorations.

### Limitations

This research was greatly hampered by extreme heterogeneity of experimental techniques, which detracted from comparability and integration of results. Studies differed widely in the nature, duration, and intensity of surface treatment regimens applied to both PEEK and titanium substrates—for example, air abrasion settings (particle size, pressure, duration), laser structuring wavelengths and intensities, and chemical etching durations were rarely controlled. Similarly, choice and formulation of bonding agents also differed widely, with some studies employing commercial primers like Visio.link or Monobond, while others employed custom or unidentified adhesive systems containing various functional monomers (e.g., UDMA, MDP, silanes), and it is difficult to identify which chemical constituents were most impactful on interfacial strength.

Further, mechanical testing protocols varied in design and execution. Whereas shear bond testing was employed in certain studies, others performed tensile pull-off or tribocorrosion testing, with enormous differences in test equipment setup, specimen dimensions, and crosshead speeds. Inability to report uniformly, e.g., load rate of application (e.g., 0.5 mm/min vs. 1 mm/min), and absence of clear calibration of measurement system prevented quantitative comparison across studies.

Certain studies also failed to report and define uniformly, e.g., peak bond strength value, failure mode (adhesive, cohesive, or mixed), and degradation upon thermocycling, which limited integration of results into a general framework. Furthermore, nearly all tests were performed in extremely controlled laboratory settings, which, while good for controlling variables, did not simulate the dynamic oral biomechanical and biochemical environment. In vivo conditions of moisture, pH cycling, microbial colonization, masticatory loading, and long-term thermal cycling were nonexistent or simulated in a limited fashion.

In the absence of long-term clinical trials or animal model studies that control for biological variables, and prosthetic aging with time, the current results must be viewed as preclinical trends and not as final clinical recommendations. This underscores the need for future work that not only standardizes experimental variables but also fills the gap between bench-top evidence and actual prosthodontic practice.

### Future recommendations and implications

A clinically focused synthesis of the literature presents a number of key considerations to maximize the adhesion strength of PEEK (Pure and Glass-reinforced) and titanium, especially for use in implant-supported prosthetic applications. Of the surface treatment methods, laser structuring and air-particle abrasion are the basic pretreatment methods consistently increasing the micromechanical retention of the PEEK–titanium interface. The treatments increase surface roughness and energy, thus enhanced wetting and penetration of the adhesive agents into the micro-irregularities. Thus, they must be considered a necessary preparatory procedure to any clinical workflow for PEEK-based prosthetic frameworks.

Of equal importance is the judicious choice of bonding agents. Adhesive systems should be chosen according to their established chemical compatibility with PEEK and titanium. Primer systems with functional monomers such as UDMA, MMA, phosphate esters, or thiophosphates are very promising since they can chemically bond the PEEK polymer chains and the titanium oxide surface and form a dual-reactive adhesive bridge. Researchers and clinicians need to take into consideration primer-based systems with established dual-surface compatibility, which can optimize bonding performance and reduce interfacial failure risks in the oral environment.

From a protocol and research design point of view, a pressing requirement is the standardization of test parameters in lab research. There needs to be standardized protocols for surface conditioning, rate of load application, dwell time, and bond testing (e.g., tensile or shear testing) to enable valid comparison between studies and materials. Harmonization will also enable meta-analytical analysis and evidence-based clinical guidance development. Also, while as much as the immediate bond strength values are reported across the globe, long-term resistance of the adhesive interfaces to intraoral conditions is not yet thoroughly examined. Long-term performance of the adhesion interface under simulated mastication and thermal cycling must be established by future research involving thermomechanical aging protocols with cyclic loading and thermocycling. In vivo animal model testing or clinical trials would also be high-level evidence for the actual performance of these adhesion methods.

## Conclusion

The observed findings showed that state-of-the-art surface treatment processes, laser structuring, and air-particle abrasion in conjunction with proprietary bonding agents enhanced considerably the adhesion strength and longevity of PEEK and reinforced PEEK composites to titanium. The implications of this work emphasize the necessity of maximizing surface and adhesive characteristics for optimized performance in bioapplications.

## Supplementary Information

Supplementary Material 1

## Data Availability

All data generated or analysed during this study are included in this published article and its supplementary information files.

## Abbreviations

PEEK: Polyetheretherketone
PEKK: Polyetherketoneketone
BioHPP: Biocompatible High-Performance Polymer (ceramic-filled PEEK composite)
Ti6Al4V: Titanium alloy composed of 6% Aluminum and 4% Vanadium
SBS: Shear Bond Strength
SEM: Scanning Electron Microscopy
EDS: Energy-Dispersive Spectroscopy
AFM: Atomic Force Microscopy
SBF: Simulated Body Fluid
MMA: Methyl Methacrylate
DMA: Dimethacrylate
PETIA: Pentaerythritol Triacrylate
HF: Hydrofluoric Acid
CAD–CAM: Computer-Aided Design – Computer-Aided Manufacturing
ISO: International Organization for Standardization
QUIN: Quality assessment tool for In vitro dental studies
SYRCLE: Systematic Review Centre for Laboratory animal Experimentation (bias tool)
VM: Vita Mark II (a brand of feldspathic ceramic)
VE: Vita Enamic (a hybrid ceramic material)
CS: Cerasmart (a composite block with dispersed fillers)
ICP-MS: Inductively Coupled Plasma Mass Spectrometry
CAD: Computer-Aided Design
CAM: Computer-Aided Manufacturing
